# Training Anesthesiology Residents to Care for the Traumatically Injured in the United States

**DOI:** 10.1213/ANE.0000000000006417

**Published:** 2023-04-14

**Authors:** Kevin P. Blaine, Roman Dudaryk, Andrew D. Milne, Tiffany S. Moon, David Nagy, Joshua W. Sappenfield, Justin J. Teng

**Affiliations:** From the * Department of Anesthesiology and Perioperative Medicine, Oregon Health and Science University, Portland, Oregon; † Department of Anesthesiology, University of Miami, Ryder Trauma Center, Miami, Florida; ‡ Trauma Anaesthesia Group, Royal London Hospital, Barts Health NHS Trust, London, United Kingdom; § Department of Anesthesiology and Pain Management, University of Texas Southwestern Medical Center, Dallas, Texas; ‖ Baptist Health, Fort Smith, Arkansas; ¶ Department of Anesthesiology, University of Florida College of Medicine, Gainesville, Florida; # Department of Anesthesia, The Permanente Medical Group, South Sacramento, California.

## Abstract

Training and education for trauma anesthesiology have been predicated on 2 primary pathways: learning through peripheral “complex, massive transfusion cases”—an assumption that is flawed due to the unique demands, skills, and knowledge of trauma anesthesiology—or learning through experiential education, which is also incomplete due to its unpredictable and variable exposure. Residents may receive training from senior physicians who may not maintain a trauma-focused continuing medical education. Further compounding the issue is the lack of fellowship-trained clinicians and standardized curricula. The American Board of Anesthesiology (ABA) provides a section for trauma education in its Initial Certification in Anesthesiology Content Outline. However, many trauma-related topics also fall under other subspecialties, and the outline excludes “nontechnical” skills. This article focuses on the training of anesthesiology residents and proposes a tier-based approach to teaching the ABA outline by including lectures, simulation, problem-based learning discussions, and case-based discussions that are proctored in conducive environments by knowledgeable facilitators.

**KEY POINTS**

- **Question**: What is the optimal structure for incorporating required American Board of Anesthesiology (ABA) content into a subspecialty curriculum for trauma?
- **Findings**: Current trauma anesthesia training required by the ABA covers a wide range of information within the trauma section in the content outlines and in other related subspecialty sections but excludes nontechnical skills.
- **Meaning**: Education of trauma anesthesiology requires implementation of a structured curriculum with case-based learning, problem-based discussion, and simulation to improve retention of knowledge, as well as, technical and nontechnical skills.

In the United States, severe traumatic injuries account for approximately 3 million emergency department admissions and 150,000 deaths per year.^1^ Major trauma is the leading cause of death for emergency department patients aged 1 to 44 years and is the third leading cause of death overall.^2^ Although operative trauma represents a minority of admissions, hospitals must maintain organizational readiness to accommodate these emergency cases when they arrive. Geographic inequalities in the distribution of accredited trauma centers across the country (45 million Americans live >60 minutes from a level 1 or level 2 center)^3^ and the lack of fellowship-trained trauma anesthesiologists means that generalist anesthesia providers will manage the bulk of these cases. Thus, it is imperative that graduates of American anesthesiology training programs are adequately trained in the perioperative evaluation and management of the critically injured.

Trauma surgeons already have concerns about the ability of general anesthesiologists to care for trauma patients. Kaslow et al^4^ conducted a survey of 450 trauma surgeons; these surgeons commented that general anesthesiologists often treat trauma patients as if they were elective patients with hemorrhage and hemodynamic instability. In the same survey, anesthesiologists reported that they believed themselves to be fully capable of caring for trauma patients “because they deal with sick patients every day,” and asserted that they did not require specific knowledge in trauma-focused resuscitation. In their conclusions, Kaslow et al noted a substantial discrepancy between perceived and actual ability to deliver appropriate care to the critically injured among anesthesiologists employed by level 1 trauma centers.

Trauma is different from elective and emergency surgical disease. It involves multisystem physiological derangement proportionate to the severity of injury plus the extent and duration of hypoperfusion. These physiological perturbations relate to neurohumoral activation, inflammatory and immunomodulatory disruptions, metabolic derangements, and hematologic embarrassment.^5^

Multifactorial trauma-associated coagulation impairment, including acute traumatic coagulopathy and resuscitation-associated coagulopathy, is recognized as trauma-induced coagulopathy. An underappreciated contributor to coagulopathy is excessive crystalloid administration; although often still used as first-line therapy for nontraumatic surgical hemorrhage, modern resuscitation research findings suggest adverse outcomes after use of crystalloids in trauma surgery.^6^ Other considerations that differ from anesthetic care unrelated to trauma include airway control, ventilator settings, and blood pressure targets. Training for resident physicians in the unique considerations of trauma anesthesia is essential to ensure that future general anesthesiologists are capable and comfortable with the management of severely injured patients.^7^

American Board of Anesthesiology (ABA) content outlines contain some trauma-focused and trauma-related topics, but these are presented as keywords and lists, which are insufficient to ensure adequate training and education for real-life trauma patients. The learning objectives within the trauma curriculum should fall within 1 of 3 domains: core knowledge (cognitive domain), technical skills (psychomotor domain), and nontechnical skills (affective domain).^8^ The delivery of teaching and training in trauma should encompass a variety of educational modalities that can be mapped to these specific domains. To provide optimal training for trauma anesthesia, training programs should ensure that all outcomes are not only covered by their curriculum but also taught via the most effective method. The educational interventions that encourage the most “doing” from learners have the greatest returns for retention of knowledge.^9^ As such, anesthesiologists in training programs should be provided with sufficient supervised clinical experience to hone the requisite skills and behaviors necessary for the progression to independent practice in trauma anesthesia.^10^ Ideally, this should be supplemented with exposure to wider aspects of trauma care.

Resident exposure to trauma is highly variable and program-specific. The Accreditation Council for Graduate Medical Education (ACGME) has recognized the need to include training in trauma management for anesthesiology/critical care fellowships,^11^ but not general anesthesiology residencies.^12^ Indeed, the ACGME Accreditation Data System Case Log no longer has a separate category for trauma and burn cases, and has now conflated trauma and burn cases into the broader category of “complex, life-threatening injuries” (minimum 20 cases).^13^ This is a vague term that could include nontrauma diseases and complications as varied as unanticipated surgical hemorrhage, intestinal ischemia, intracranial hypertension, or ascending aortic dissection. Furthermore, hospital trauma volume varies across residency programs, as do types of injuries. Consequently, residents are taught to anesthetize trauma patients on a “first come, first served” basis, learning from the random assortment of cases that they happen to encounter during their training. The limitations of trauma education are compounded when residents are taught by attendings who themselves may not have undergone rigorous training for trauma in their own residencies. A structured curriculum for trauma anesthesiology during residency would mitigate the variable exposure to trauma cases.

There is an urgent need to standardize education in trauma resuscitation. To that end, we reviewed the content outlines for the ABA to identify existing topics related to trauma. We then explored didactic methods that have shown success in other medical education venues, including lectures, simulation, problem-based learning, and case-based learning. Finally, we have proposed a structure for a potential trauma curriculum.

## METHODS

Requirements for primary board certification, as included in the ABA Initial Certification Content Outline,^14^ Objective Structured Clinical Examination (OSCE) Content Outline,^15^ and Maintenance of Certification in Anesthesiology (MOCA) Content Outline,^16^ were reviewed independently (authors J.W.S. and J.J.T.). The reviewers categorized each requirement as related to, or not related to, the anesthetic management of trauma patients. Each reviewer independently examined the content outlines and determined which elements were important for trauma care training in residency. Disagreements between the reviewers were adjudicated by a third reviewer (R.D.), who made the final determination on whether the content was relevant. The final agreed-upon content from the Initial Certification Content Outline can be found in Appendix 1, the final content from OSCE Content Outline can be found in Appendix 2, and the final content from MOCA Content Outline can be found in Appendix 3.

The ABA content outline subsections on trauma and burns covered trauma material for both initial certification and MOCA. As seen in Supplemental Digital Content 1, Table 1, http://links.lww.com/AA/E225, and Table 1, a significant amount of content outside those subsections was also considered to be trauma related.

**Table 1.** Representative Content Deemed Trauma Related From the Maintenance of Certification in Anesthesiology Content Outline Which Is Not Found in Section V.I Trauma Anesthesia and XI.C.7 Trauma and Burns

Heading	General theme	Specific examples
Fundamental topics	Monitoring	Point-of-care ultrasound, coagulation, etc
	Perioperative management of patients with chronic disease states	Spinal cord injury, etc
Clinical sciences	Organ-based	Chest trauma, tamponade, cardiac contusion, etc
	Hematologic	Transfusions, citrate intoxication, DIC, fibrinolysis, MTP etc
	Critical care anesthesia	Brain death, spinal cord compromise, multiorgan failure, etc
	Neuroanesthesia	Cerebral blood flow, ICP, TBI, etc
	Cardiovascular	Awareness, aortic clamping, etc
	Regional anesthesia	Truncal regional anesthesia, etc
	Burns	Airway management, complications, resuscitation, etc
	Disaster management	Biologic, chemical, nuclear, etc
Pharmacology	Hematologic	Anticoagulants, blood substitutes, coagulation factor replacement therapy, etc
	Neurologic	Anticonvulsants, diuretics, steroids, etc
Ethics, medicolegal		DNR, end-of-life issues, Jehovah’s witness, etc

Abbreviations: DIC, disseminated intravascular coagulation; DNR, do not resuscitate; ICP, intracranial pressure; MTP, massive transfusion protocol; TBI, traumatic brain injury.

The OSCE content outline did not have a trauma subsection. However, additional content topics included point-of-care ultrasound and echocardiography (Table 2). Point-of-care ultrasound and echocardiography could be found in all 3 content outlines.

**Table 2.** Representative Content Deemed Trauma Related From the Objective Structured Clinical Examination Content Outline

Topic	Examples
Point-of-care ultrasound	Standard views of the heart
	Lung anatomy and artifacts
	Assessment for abdominal free fluid
	Assessment of gastric content and volume

Other themes in the initial certification content outline included: hypothermia; understanding of anatomy and its implications for injuries; acquired coagulopathies and medications that affect coagulation; initiation of resuscitation and potential pitfalls; transfusion-related complications; airway management with consideration for cervical spine injuries; management of traumatic brain injury; and management of shock. Significant overlap was seen in the content outline for MOCA. Other topics mentioned include: toxicology, drowning, aortic cross-clamping, Jehovah’s witnesses, and determination of brain death.

## DISCUSSION

The core of trauma anesthesia includes knowledge that is generic to anesthesia (such as ventilator management and pharmacology), but nuanced to reflect the specific physiological and technical considerations unique to trauma patients. Key aspects of adult learning are self-direction and internal motivation, which faculty can facilitate by providing residents with the means to efficiently find resources that cover these core principles. This can be achieved by generating a local list of learning materials that are made available to residents before the start of their trauma anesthesia focus. This list could include informative journal articles, textbook chapters, review of patient radiological images, podcasts, recorded lectures, reference cases, and simulation.

**Figure.**
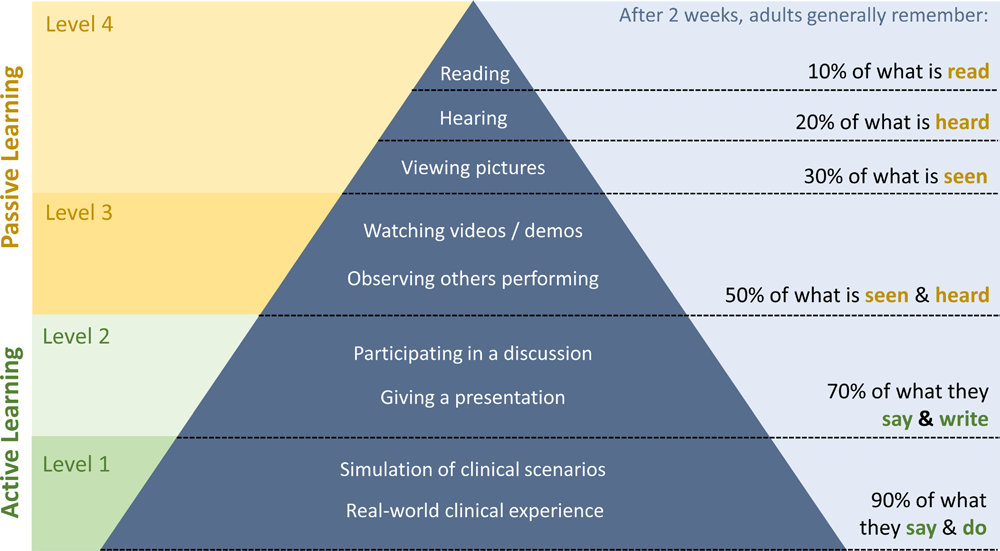
A modification of the cone of experience (Dale, 1969).^9^

It is insufficient to provide these resources and assume residents will be able to consolidate their knowledge via clinical application. The Figure demonstrates the impact of various learning methods on knowledge retention among adults and underscores the importance of delivering active learning opportunities to residents. Level 1 educational interventions are those that encourage >70% retention of the information covered. Level 2 educational interventions encourage 50% to 70% retention of the information covered. Levels 3 and 4 interventions encourage 30% to 50% and <30%, respectively. An educational curriculum should form the backbone to a series of educational sessions, facilitated by faculty but delivered by residents by means of active learning techniques while prioritizing interventions that have the highest retention of knowledge. These include problem-based learning discussion (PBLD), case-based discussion (CBD), and resident presentations at departmental meetings. The unifying objective is for the learner to apply the discrete pieces of medical knowledge gained through their prior learning into novel scenarios.

### Level 1 Educational Interventions

A model of this integrated trauma education may be found in the United Kingdom. Part of the approach to anesthesia training in the United Kingdom is the use of structured learning events (SLEs)^17^ for each rotation across the curriculum, such as for cardiac anesthesia, pediatric anesthesia, etc. SLEs include (1) direct observation of practical skills in which a consultant (attending) assesses the resident’s ability to perform specific procedures, and (2) Anaesthesia Clinical Evaluation eXercises, in which a consultant directly assesses a resident’s performance across an episode of patient care. SLEs provide a measurable means of documenting knowledge acquisition and competency progression. They also ensure that residents’ knowledge and clinical practice are appropriately challenged, and learning opportunities within clinical practice are maximized. An educational approach similar to the British SLEs can be applied to residency training in the United States. Curricula can be developed at each training site utilizing real-world cases and local expertise to better integrate existing knowledge to support trauma training. Feedback is an essential component of much of the practical and behavioral components of medical curricula. Feedback should be timely, structured, and learner-centric^18^ and should foster a learning culture in which constructive feedback is the norm, and residents are encouraged to seek feedback.^19^

It can be challenging to provide immediate feedback during a busy trauma shift. While postevent CBDs provide one way to reflect on performance, another useful technique is the after-action review (AAR) or postevent debrief.^20,21^ One study indicated that effective clinical debriefings can be achieved in 10 minutes.^22^ This study used the Summary of event, Things that went well, Opportunities for improvement, and Points of action (STOP) structure. While these should not replace timely, individual feedback, AARs should encourage residents to reflect on their own performance within the team and help inform a constructive discussion at a more convenient time.

Level 1 interventions include simulation of clinical scenarios. Simulation (both high fidelity and low fidelity) is an existing option that can contribute greatly to the development of cognitive and psychomotor skills for the high-stress environment of trauma and resuscitation. The use and capabilities of technologically enhanced simulation training have expanded greatly in the last 2 decades, and it is now firmly entrenched in the curricula of many acute specialties.^23–25^ Simulation training allows the integration of clinical, procedural, and nontechnical skills in a facsimile of the actual clinical environment, in which residents can be exposed to the pressure of high-stake patient care without the impact of real-world errors. Mannequin fidelity varies between simple plastic representations of patients with no functionality to those with fully manipulatable physiology and pathophysiology, which may include features such as pneumothoraxes and wounds that do not stop bleeding until a tourniquet has been applied to an adequate pressure. Programs should determine what learning objectives they wish to cover using simulation-based learning; if it is solely for the development of nontechnical skills, they would not require a costly, high-fidelity model. Of note, there have been calls for standards in medical simulation equipment following an aviation incident that was blamed on a substandard simulator that reinforced negative learning.^26^ The critical requirement in simulation-based education is the presence of faculty who are trained and experienced in debriefing and providing effective feedback.^27–29^ Simulation allows for the creation of a variety of case scenarios on demand and in a safe environment.^29,30^ In trauma settings, simulation training is an invaluable tool to develop multidisciplinary preparedness for massive casualty incidents, which are rare and unpredictable.

Simulation has been shown to be effective in the education of residents for care of the traumatically injured. Trauma education modalities include simulated patients, task trainers, computerized patient simulators, and virtual reality.^28,29^ Simulated patients, used in Advanced Trauma Life Support (ATLS), allow for assessment of management skills, as opposed to cognitive assessment of a written examination.^29^ Simulator training has been used to reinforce concepts taught in ATLS.^30^ Mannequins can simulate multiple examination findings and allow for procedures such as airway management, chest tube insertion, peritoneal lavage, and focused abdominal sonography for trauma,^28,29,31,32^ and research has shown that these skills are transferable to care on humans.^29^ Simulation has also been used to assess whether residents have acquired technical and nontechnical skills after training.^28,31,32^ Not only has simulation been proven to help with training skills, but also has been useful in detecting deficiencies in training, such as stabilizing children, discovering deviations in airway management, and evaluating a deteriorating intubated trauma patient.^28,29^ There are limitations to using mannequins; they may have differences in anatomy and the forces applied during laryngoscopy may vary.^29^

Simulation also allows for team training, which builds on nontechnical skills, such as decision-making, developing situational awareness, and building interpersonal communication.^29^ Another benefit of team training is it has allowed for shorter time intervals between initiation of care to various end points.^29^ Steinemann et al^33^ have developed and validated a Trauma Nontechnical Skills Scale (T-NOTECHS) score to evaluate clinical performance of nontechnical skills during trauma resuscitations. It focuses on the 5 behavioral domains: leadership, cooperation and resource management, communication and interaction, assessment and decision-making, and situational awareness/coping with stress. The advantage of this assessment is its utility in both a simulated environment and a real clinical scenario, either live or as a video recording.^33,34^ Another similar validated assessment for team training in trauma resuscitation is the Trauma Team Performance Observation Tool.^35^ Higher scores in nontechnical skills on these assessments have shown a decrease in time to disposition, greater completion of tasks, and reduction of delays in patient management.^33,36^ Training for proficiency in nontechnical skills has been shown to decrease time to moving the patient out of the trauma bays in some studies,^37^ and has also been shown to decrease time to multiple discrete points over the course of resuscitation.^33,35^ In addition to improved task completion and identification of safety threats, team training even has the potential to improve interprofessional collaboration within an institution.^38^

A notable weakness in team training simulation is the lack of an objective assessment.^28,29^ However, training may improve interrater reliability. Barriers to simulation include clinical demands, lack of funding, perceived lack of evidence supporting efficacy, limited expertise in debriefing, and paucity of leadership.^37^ Another perceived barrier to team training simulation is the cost. Most of the cost lies in the initial investment, and costs per training decreased significantly when up to 70 to 80 teams of participants were trained annually.^39^ The estimated cost to raise a participant’s T-NOTECHS score by 1 point was €427.^39^ In one study, the only barrier that was associated with the number of simulations performed was lack of buy-in from surgeons.^37^

### Level 2 Educational Interventions

The next level of interventions involves participation by the learner in the absence of the hands-on component: CBDs and PBLDs. These are of particular importance when simulations and clinical experience are unavailable. CBDs are discussions in which a resident selects a case that they personally performed to present to an attending to ensure that the resident has appreciated the situation. CBDs allow for assessment of knowledge and the rationale for the learner’s approach. The goal of a CBD is to promote the practice of reflective learning among residents. Key to this process is a willingness to scrutinize past performances with input from faculty skilled in structured, constructive feedback. CBDs can be similar to the traditional “morbidity and mortality” format, where adverse events are reviewed publicly to detect deficiencies or challenges to care. However, a CBD need not involve an adverse event because the focus is on learning and retaining skills, not safety. They also do not need to be public, and instead should be in a safe learning environment, especially when residents reassess a situation that was beyond their abilities or where the outcome was unfavorable. The intimacy of a one-on-one or small group discussion may even generate more thoughtful discourse.

A CBD may take the form of the resident presenting the background, key findings, and anesthetic management of a challenging trauma case with the faculty member identifying key learning points as the case presentation progresses. These learning points may include talking through the basis of physiological phenomena encountered, exploring alternative means of managing specific aspects of the case, and highlighting common pitfalls in trauma management. The latter is especially useful, as it can present an opportunity to discuss shortfalls in resident performance during the case; such discussion serves to compound learning and ensure progression of clinical capability when delivered in an objective, sensitive fashion. A friendly, cooperative CBD (as opposed to an antagonistic and overly critical process) can help develop reflective clinical practice and lifelong attention to self-improvement.

Another teaching method that learners in the United States may be more familiar with is PBLD, where learners review a fictional or semifictional scenario with a facilitator. PBLDs typically take the form of a stem narrative composed of a clinical scenario with subsequent open questions such as, “how would you approach the airway?” or “what is your approach to vasopressor management?” PBLDs are especially useful for trauma as they can be generated well in advance, standardized for all residents, and mapped specifically to core topics.

For example, a local site may wish to write a PBLD based on a particularly challenging anesthetic that involved several different trauma domains. Suppose the PBLD writer had recently managed a blunt trauma patient with a simultaneous abdominal injury and injury to the trachea. Recalling these details, and the successful and unsuccessful attempts that were made, a standardized scenario can be created where residents removed from the situation can talk through their management priorities. Key events in this case can generate open-ended questions. If necessary, the writer could take some literary license to include decision points that were not encountered in the original event. A library of such PBLD stems, and questions could then be generated to ensure that the material never becomes stale or rote. Cases can be reviewed multiple times if the resident wishes to revise or reconsider approaches to difficult cases or if an educator decides to tweak details to explore other decision pathways. Complexity can be expanded from isolated injury cases (eg, only airway trauma) to multiple injuries (eg, airway and chest trauma), and to patients with more complex medical history (eg, atrial fibrillation on dabigatran). PBLDs can even be shared between sites, although local or personal knowledge of a difficult case (particularly from a knowledgeable faculty) will provide a more authentic and memorable experience for the learner. Guidelines for PBLD development have been previously published, and the authors recommend resources from the Society for Pediatric Anesthesia, American Society of Anesthesiologists, and peer-reviewed literature.^40–42^

PBLDs and CBDs are similar in that both are learner-centric modalities that enable residents to solve problems with active knowledge acquisition and collaborative learning within small groups, while retaining high relevancy to clinical practice.^42^ They are a crucial supplement for education when simulation and clinical experience are unavailable or lacking.

### Level 3 and 4 Educational Interventions

The third and fourth levels of interventions involve modalities where learners take a more passive approach to learning. This includes shadowing clinicians in different clinical environments, watching web-based videos, and participating in discussion forums. Also included in these levels are traditional lecture-based didactics and reading textbooks. Younger generations may utilize newer modalities such as podcasts and social media to facilitate their learning experience. While these modalities do not yield the highest retention of information, they can be useful to supplement other more hands-on and active learning strategies as described above.

### Skillsets of Anesthesiologists Outside of US Civilian Care

Trauma anesthesiologists often deliver care beyond conventional perioperative roles. Among US physicians, these roles are predominantly military or humanitarian in nature; they often involve practice in austere environments with limited resources and typically with more responsibilities than hospital-based anesthesiologists. Médecins Sans Frontières (Geneva, Switzerland) recently described a predeployment, blended-learning module for residents covering supplementary aspects, such as disaster medicine, safety and security, and infectious disease.^43^ Military trauma anesthesiologists require further training to provide damage control resuscitation in prolonged field care settings and additional specialist knowledge of chemical, biological, radiological, and nuclear exposure management.^44^

Outside the United States, anesthesiologists and emergency physicians comprise most physicians delivering prehospital care. These expanded roles require further specialist knowledge and training beyond that of operative trauma anesthesiology, which creates opportunities for complementary skills, such as advanced resuscitative interventions and fracture management. Anesthesiologists further contribute to the care of patients with circulatory collapse requiring mechanical support as part of extracorporeal membrane oxygenation teams.^44^ In the United Kingdom and Canada, anesthesiologists frequently perform the role of trauma team leader. A key skill taught to these trainees is the rapid formation of high-performing, ad hoc teams using nontechnical cognitive skills honed by multiprofessional simulation training.^45^

Effective pain management beyond the immediate perioperative period is associated with improvement across several outcome measures including a reduced incidence of chronic pain.^44^ Specialist anesthesia input via clinical and organizational leadership during the entirety of trauma patients’ timelines, from the prehospital phase through to critical care and beyond, is integral to improving outcomes. Parallel yet distinct knowledge and training are required to meet an interfacility transfer to specialist trauma hospitals. In many countries, anesthesiologists are key to the coordination and clinical management of these complex and hazardous periods in trauma patients’ timelines.^46^

## CONCLUSIONS

Knowledge and experience in trauma anesthesia are necessary for general anesthesiologists graduating from residency programs because trauma is a surgical disease that many graduates will be expected to manage in their routine practice. Trauma anesthesiology demands both broad knowledge and a unique subset of technical and nontechnical skills. Using the ABA content outline as a starting point, we discuss implementation of a tiered approach to education of the anesthesiology resident. As rated by retention rates, clinical and simulation experience have the highest yield in learning and allow for work-based assessments. This is further augmented by the subsequent levels of education, including PBLDs, CBDs, and AARs, followed by lectures, didactics, podcasts, and books. Particularly in places that do not have access to clinical or simulation scenarios, we recommend using the next level of available resources to improve care of our traumatically injured patients.

A training standard should be developed to provide a framework for assessment of future curricula. Currently, the requirements only include emergency cases (which may or may not be trauma) and a knowledge base that is assessed using multiple-choice questions. Future scholarly work is needed to develop an evidence-based curriculum for trauma anesthesia practice.

## DISCLOSURES

**Name:** Kevin P. Blaine, MD, MPH, FASA.

**Contribution:** This author helped with planning of the studies and manuscript, analysis of the results, writing the original manuscript, and editing of the final manuscript.

**Name:** Roman Dudaryk, MD.

**Contribution:** This author helped with planning of the studies and manuscript, analysis of the results, writing the original manuscript, and editing of the final manuscript.

**Name:** Andrew D. Milne, MBChB, FRCA, DMCC.

**Contribution:** This author helped with planning of the studies and manuscript, analysis of the results, writing the original manuscript, and editing of the final manuscript.

**Name:** Tiffany S. Moon, MD, FASA.

**Contribution:** This author helped with planning of the studies and manuscript, analysis of the results, writing the original manuscript, and editing of the final manuscript.

**Name:** David Nagy, MD.

**Contribution:** This author helped with planning of the studies and manuscript, analysis of the results, writing the original manuscript, and editing of the final manuscript.

**Name:** Joshua W. Sappenfield, MD.

**Contribution:** This author helped with planning of the studies and manuscript, analysis of the results, writing the original manuscript, and editing of the final manuscript.

**Name:** Justin J. Teng, MD.

**Contribution:** This author helped with planning of the studies and manuscript, analysis of the results, writing the original manuscript, and editing of the final manuscript.

**This manuscript was handled by:** Richard P. Dutton, MD.

## Supplementary Material



## Appendix 1. Content Outline for Initial Certification in Anesthesiology With Nontrauma Relevant Material Omitted

Adapted from: The American Board of Anesthesiology. Initial Certification in Anesthesiology. 2021. Accessed January 3, 2022. https://www.theaba.org/pdfs/Initial_Certification_Content_Outline.pdf

The following content was selected from the American Board of Anesthesiology Content Outline. The original content outline numbering is retained for easy reference, and is thereby discontinuous.

### I. BASIC TOPICS IN ANESTHESIOLOGY

#### A. Basic Sciences

1. Anatomy
  a. Topographical Anatomy as Landmarks
    1) Neck: Cricothyroid Membrane, Internal and External Jugular Veins, Thoracic Duct, Carotid and Vertebral Arteries, Stellate Ganglion, Cervical Spine Landmarks (Vertebra Prominens, Chassaignac’s Tubercle), Hyoid Bone, Superficial Cervical Plexus
    2) Chest: Pulmonary Lobes, Cardiac Landmarks, Subclavian Vein
  b. Radiological and Ultrasound Anatomy
    Chest (Including CT and MRI)
    Brain and Skull (Including CT and MRI)
    Spine (Cervical, Thoracic, Lumbar), Including CT and MRI
    Neck (Including Doppler Ultrasound for Central Venous Access)
    Abdominal wall
    Extremities
2. Physics, Monitoring, and Anesthesia Delivery Devices
  g. Physics of Anesthesia Machine/Breathing System
    3) Characteristics
      e) Toxicity: Carbon Monoxide
  h. Monitoring Methods
    Oxygen: Co-oximetry
  i. Instrumentation
    Fluid Warmers, Autotransfusion Devices
4. Pharmacology
  b. Anesthetics — Gases and Vapors
    3) Effects on Central Nervous System

#### B. Clinical Sciences: Anesthesia Procedures, Methods, and Techniques

1. Evaluation of the Patient and Preoperative Preparation
  d. Preparation for Anesthesia/Premedication
    6) NPO and Full Stomach Status; Implications for Airway Management, Choice of Anesthesia Technique and Induction of Anesthesia; Gastric Emptying Time; Preoperative; Full Stomach and Induction of Anesthesia; Practice Guidelines for Preoperative Fasting
    10) Oral Anticoagulants and Antiplatelet Agents
3. General Anesthesia
  c. Airway Management
    1) Assessment/Identification of Difficult Airway: Anatomic Correlates, Mallampati Classification, Range of Motion
    2) Techniques for Managing Airway: Awake vs. Asleep, Use vs. Avoidance of Muscle Relaxants, Drug Selection, Retrograde Intubation Techniques, ASA Difficult Airway Algorithm
    3) Devices: Flexible Fiberoptic, Rigid Fiberoptic, Transillumination, Laryngoscope Blades, Alternative Intubating Devices, Video Laryngoscopes
    4) Alternatives and Adjuncts: Laryngeal Mask Airway (Traditional and Modified), Esophageal Obturator Airways, Occlusive Pharyngeal Airways
    5) Transcutaneous or Surgical Airway: Tracheostomy, Cricothyroidotomy, Translaryngeal or Transtracheal Jet Ventilation
    6) Endobronchial Intubation: Double-Lumen Endobronchial Tubes; Bronchial Blockers, Placement and Positioning Considerations, Postoperative Considerations
    7) Intubation and Tube Change Adjuncts: Bougies, Jet Stylettes, Soft and Rigid Tube Change Devices; Complications
6. Common Complications
  c. Temperature
    1) Hypothermia: Etiology, Prevention, Treatment, Complications (Shivering, O2 Consumption), Prognosis

#### C. Organ-Based Basic and Clinical Sciences

1. Central and Peripheral Nervous System
  a. Physiology
    1) Brain
      c) Cerebral Blood Flow
        (1) Effect of Perfusion Pressure, Ph, PaCO2, PaO2, and Cerebral Metabolic Rate for O2 (CMRO2); Inverse Steal; Gray vs. White Matter
        (2) Autoregulation: Normal, Altered, and Abolished
        (3) Pathophysiology of Ischemia/Hypoxia: Global vs. Focal, Glucose Effects, Effects of Brain Trauma or Tumors
  b. Anatomy
    Meninges: Epidural, Subdural, and Subarachnoid Spaces
2. Respiratory System
  a. Physiology: Lung Functions and Cellular Processes
    3) Ventilation: Perfusion
      a) Distribution of Ventilation
      b) Distribution of Perfusion, Zones, Hypoxic Pulmonary Vasoconstriction
      c) Alveolar Gas Equation
    4) Diffusion
      Definition, Pulmonary Diffusion Capacity
      Apneic Oxygenation, Diffusion Hypoxia
    5) Blood Gas
      Systemic Effects of Hypercarbia and Hypocarbia
      Systemic Effects of Hyperoxia and Hypoxemia
      Basic Interpretation of Arterial Blood Gas
3. Cardiovascular System
  a. Physiology
    3) Venous Return
      a) Vascular Compliance/Venous Capacitance; Controlling Factors
      b) Muscle Action; Intrathoracic Pressure; Body Position
      c) Blood Volume and Distribution
    4) Blood Pressure
      Systolic, Diastolic, Mean, and Perfusion Pressures
      Intracardiac, Pulmonary, Venous
      Systemic and Pulmonary Vascular Resistance, Viscosity
      Baroreceptor Function
    5) Microcirculation
      Capillary Diffusion; Osmotic Pressure, Starling’s Law
      Pre-Post Capillary Sphincter Control
      Viscosity; Rheology
    6) Regional Blood Flow and Its Regulation
      Cerebral and Spinal Cord
      Coronary
      Pulmonary
      Renal
      Splanchnic: Hepatic
      Muscle and Skin
      Uterine and Placental
6. Hematologic System
  a. Pharmacology
    1) Anticoagulants, Antithrombotics, and AntiPlatelet Drugs
      a) Mechanism of Action
      d) Monitoring of Effects
  b. Transfusions
    Indications
    Blood Preservation, Storage
    Blood Filters and Pumps
    Effects of Cooling and Heating; Blood Warmers
    Blood Components, Volume Expanders
    Preparation for Transfusion: Type and Cross, Type and Screen, Uncrossmatched Blood, Autologous Blood, Designated Donors
    Synthetic and Recombinant Hemoglobins
  c. Reactions to Transfusions
    Febrile
    Allergic
    Hemolytic: Acute and Delayed
  d. Complications of Transfusions
    1) Infections: Hepatitis, Human Immunodeficiency Virus (HIV), Cytomegalovirus (CMV), Others
    2) Citrate Intoxication
    3) Electrolyte and Acid-Base Abnormalities
    4) Massive Transfusion: Coagulopathies, Hypothermia
    5) Pulmonary
      a) Transfusion-Related Acute Lung Injury
      b) Transfusion-Related Circulatory Overload
    6) Immunosuppression

### II. ADVANCED TOPICS IN ANESTHESIOLOGY

#### A. Basic Sciences

1. Physics, Monitoring, and Anesthesia Delivery Devices
  a. Monitoring Methods
    3) Brain and Spinal Cord Function: Intracranial Pressure
  b. Instrumentation
    Echocardiography: Technical Aspects, Complications
    Coagulation Monitors
    Ultrasound-Guided Placement of Vascular Catheters (Arterial, Central Venous) and Nerve Blocks
    Point-of-Care Ultrasound (POCUS): Lung, IVC, Bladder, Gastric

#### B. Clinical Sciences: Anesthesia Procedures, Methods, and Techniques

1. Regional Anesthesia
  f. Implications and Management of Anticoagulants and Platelet Inhibitors
2. Special Techniques
  Extracorporeal Support

#### C. Organ-Based Advanced Clinical Sciences

1. Central and Peripheral Nervous Systems
  a. Physiology
    2) Intracranial Pressure
      a) Brain Volume, Elastance and Compliance
      b) Increased ICP, Herniation
  d. Clinical Science
    1) Central Nervous System
      b) Coma
        (1) Glasgow Coma Scale, Management of Traumatic Brain Injury
      d) Paraplegia, Quadriplegia, Spinal Shock, Autonomic Hyperreflexia
        Airway Management in the Patient with Cervical Spine Disease
      f) Special Problems of Anesthesia for Neurosurgery
        Increased Intracranial Pressure:
        Anesthetic and Ventilatory Effects on Cerebral Blood Flow and Metabolism
        Fluid Management: Hypertonic vs. Isotonic Saline vs. Balanced Salt Solutions
2. Respiratory System
  d. Clinical Science
    1) Respiratory System
      a) Obstructive Disease
        (1) Upper Airway: Traumatic
        (2) Tracheobronchial: Traumatic
      b) Restrictive Disease
        Musculoskeletal: Chest Trauma
3. Cardiovascular System
  a. Normal Anatomy of Heart and Major Vessels
    1) Echocardiographic Heart Anatomy: Chambers, Valves, Great Vessels, Pericardium, Basic Transesophageal Echocardiography (TEE) Views
    5) Cardiac Tamponade and Constrictive Pericarditis
      a) Etiology
      b) Diagnosis; TEE, PA Catheter
      c) Anesthetic Management
    7) Pulmonary Embolism
      Etiology: Blood, Air, Fat, Amniotic Fluid
      Diagnosis, TEE findings
      Treatment; Acute, Preventive
    9) Shock States: Anesthetic Management of Patient in Shock
6. Hematologic System
  a. Clinical Science
    1) Hematologic Disorders
      a) Diseases of Blood
        (3) Clotting Disorders
          (d) Fibrinolysis
          (e) Pharmacologic: Anticoagulants and Antagonists
  b. Advanced Transfusion Therapy: Indications, Reactions
    Febrile
    Allergic
    Hemolytic: Acute and Delayed
  c. Complications of Transfusions
    1) Infections: Hepatitis, Human Immunodeficiency Virus (HIV), Cytomegalovirus (CMV), Others
    2) Citrate Intoxication
    3) Electrolyte and Acid Base Abnormalities
    4) Pulmonary
      a) Transfusion-Related Acute Lung Injury
      b) Transfusion-Related Circulatory Overload
    5) Immunosuppression
  d. Transfusion Therapy for Massive Hemorrhage
    1) Massive Transfusion Protocol
      a) Ratios of RBC and Plasma
      b) Use of Uncrossmatched Products
      c) Adjuvant Therapies: Antifibrinolytics, Calcium, etc.
    2) Coagulopathy of Hemorrhagic Shock

#### D. Clinical Subspecialties

9. Trauma Anesthesia
  a. Massive Trauma
    1) Evaluation of the Trauma Patient
    2) Resuscitation from Hemorrhagic Shock
  b. Management of Traumatic Brain Injury
  c. Burn Management
  d. Mass Casualty, Disaster Management, and Preparedness
  e. Chemical and Biological Warfare
12. Critical Care
  a. Shock States
    1) Etiology, Classification, Pathophysiology
    2) Septic Shock and Life-Threatening Infection
    a) Sequential (Sepsis Related) Organ Failure (SOFA) Assessment Score
    3) Systemic Inflammatory Response Syndrome
    4) Multiple Organ Dysfunction Syndrome
  c. Drowning

#### E. Special Problems or Issues in Anesthesiology

2. Organ Donors: Pathophysiology and Clinical Management
  a. Circulatory
  b. Endocrine
  c. Fluid and Electrolyte Management
  d. Brain Death Criteria

## Appendix 2. Content Outline for the Objective Structured Clinical Examination (OSCE) Content Outline With Nontrauma Relevant Material Omitted

Adapted from: American Board of Anesthesiology. Applied Exam: Objective Structured Clinical Examination (OSCE) Content Outline. https://theaba.org/pdfs/OSCE_Content_Outline.pdf. Accessed January 3, 2022.

The following content was selected from the American Board of Anesthesiology Content Outline. The original content outline numbering is retained for easy reference, and is thereby discontinuous.

### B. TECHNICAL SKILLS

2. Interpretation of echocardiograms and surface ultrasound of lung (Interpret basic transthoracic or transesophageal, lung and pleura images relevant to anesthesia practice). The successful candidate will be able to identify the view, identify relevant anatomy, make qualitative diagnostic assessments, and provide treatment recommendations for scenarios chosen from among the following areas:
  a. Biventricular function and wall motion
  c. Volume status assessment — hypovolemia and response to volume therapy
  d. Pulmonary emboli
  e. Air emboli
  g. Pericardial effusions
  h. Aortic dissection
  i. Pleural effusion
  j. Pneumothorax
  k. Pulmonary edema
3. Application of ultrasonography (Identify relevant normal anatomy using ultrasonography). Candidates who take the virtual APPLIED Exam will not participate in this skill station. The successful candidate will identify the relevant anatomy using an ultrasound probe with a simulated patient and, where applicable, may be asked to demonstrate simulated needle placement technique for scenarios chosen from among the following procedures:
  c. -Point-of care ultrasound
    Heart (testing to start in 2022)

• Parasternal Long Axis
• Parasternal Short Axis (Left Ventricle Midpapillary)
• Apical Four Chamber
• Subcostal Four Chamber
• Subcostal IVC View

ii. Lung (testing to start in 2022)
  • Pleura
  • Diaphragm
  • Artifacts (A-lines, B-lines)
iii. Abdomen (testing to start no earlier than 2022)
  • Right upper quadrant (assessment for free fluid)
  • Left upper quadrant (assessment for free fluid)
  • Pelvis (assessment for free fluid)
  • Gastric (assessment of content and volume)

## Appendix 3. Content Outline for the Maintenance of Certification in Anesthesiology (MOCA) With Nontrauma Relevant Material Omitted

Adapted from: American Board of Anesthesiology. Maintenance of Certification in Anesthesiology (MOCA) Content Outline. https://theaba.org/pdfs/MOCA_Content_Outline.pdf. 2022. Accessed January 3, 2022.

The following content was selected from the American Board of Anesthesiology Content Outline. The original content outline numbering is retained for easy reference, and is thereby discontinuous.

### I. FUNDAMENTAL TOPICS IN ANESTHESIOLOGY

#### C. Monitoring

2. Advanced Physiologic
  TAGS:
  Point-of Care Ultrasound
3. Intraoperative Blood Monitoring
  TAGS:
  Coagulation
  Point-of Care Laboratory Testing

### III. CLINICAL SCIENCES: ANESTHESIA PROCEDURES, METHODS AND TECHNIQUES

#### B. Perioperative Management of Patients With Chronic Disease States

1. Central and Peripheral Nervous System
  TAGS:
  Spinal Cord Injury

### IV. Organ-Based Basic and Clinical Sciences

#### B. Respiratory System

4. Clinical Science
  TAGS:
  Chest Trauma

#### C. Cardiovascular System

4. Clinical Management of Disease States
  TAGS:
  Cardiac Tamponade and Constrictive Pericarditis

#### F. Hematologic System

3. Pharmacology
  TAGS:
  Anticoagulants and Antagonists
  AntiPlatelet Drugs
  Blood Substitutes
  Coagulation Factor Replacement Therapy
  Erythropoietin
  Immunosuppressive and Anti-Rejection Drugs
  Iron Therapy
4. Clinical Science
  TAGS:
  Alternatives to Transfusion
  Anemias
  Autologous Blood Donation
  Blood Products
  Citrate Intoxication
  Congenital and Acquired Factor Deficiencies
  Disseminated Intravascular Coagulation
  Fibrinolysis
  Hemoglobinopathies
  Hypothermia: Effects
  Infections: Cytomegalovirus, HIV, Hepatitis
  Massive Transfusion: Acquired Coagulopathy
  Massive Transfusion Protocol
  Porphyrias
  Thrombocytopenia and Thrombocytopathy
  Transfusion Complications Including Transfusion-Associated Circulatory Overload and Transfusion-Related Acute Lung Injury
  Transfusion Indications
  Transfusion: Infection Risks

### I. TRAUMA ANESTHESIA

1. Primary Survey and Resuscitation
  TAGS:
  Emergency Airway
  Hemorrhagic Shock
  Initial Evaluation
  Mass Casualty Response
  Penetrating vs. Blunt Trauma
  Prehospital and Emergency Medical Services
  Team Function in Trauma
  Triage
2. Secondary Survey and Stabilization
  TAGS:
  Fluid Management
  Hemostatic Resuscitation
  Hypothermia
  Massive Transfusion
  Monitoring
  Protection of Cervical Spine
3. Organ System Trauma
  TAGS:
  Abdominal Trauma
  Aortic and Vascular Trauma
  Orthopedic and Soft Tissue Trauma
  Spinal Cord
  Thoracic Trauma
  Traumatic Brain Injury
  Traumatic Coagulopathy
4. Thermal and Electrical Injury
  TAGS:
  Airway Management
  Carbon Monoxide/Carboxyhemoglobin
  Fluids and Electrolytes
  Inhalation Injury
  Non-depolarizing Muscle Relaxants
  Succinylcholine Use
  Vasoconstrictor Effects and Complications
5. Special Considerations in Trauma Anesthesia
  TAGS:
  Decontamination
  Exposure/Hypothermia
  Near Drowning
  Nuclear, Biological, and Chemical Injury
  Pain Management
  Pediatric Trauma

#### L. Critical Care Anesthesia

1. Central Nervous System Dysfunction
  TAGS:
  Brain Death
  Brain Trauma
  Spinal Cord Compromise
  Stroke: Ischemic or Hemorrhagic
  Subarachnoid, Epidural Bleed
7. Hematologic Dysfunction
  TAGS:
  Anticoagulation/Antiplatelet/Antifibrinolytic Therapy
  Coagulopathy: Disseminated Intravascular Coagulation, Consumptive, Dilutional
  Transfusion Therapy
11. Additional Critical Care Topics
  TAGS:
  Multi-Organ Failure
  M. Neuroanesthesia
1. Clinical Science
  TAGS:
  Factors Impacting Cerebral Blood Flow
  Factors Impacting Intracranial Pressure
  Factors Impacting Neuronal Function
2. Pharmacology
  TAGS:
  Anesthetics
  Anticonvulsants
  Diuretics
  Fluid Management
  Steroids
3. Clinical Management of Disease States
  TAGS:
  Traumatic Brain Injury
4. Special Considerations in Neuroanesthesia
  TAGS:
  Cerebral Blood Flow
  Cerebral Herniation
  Cerebral Ischemia
  Cerebral Vasospasm
  Interventional Radiology
  Intraoperative MRI
  Neurophysiologic Monitoring
  Patient Positioning
  Seizures
  Spinal Drains
  Venous Air Embolism
  Ventriculostomy

#### N. Thoracic Anesthesia

3. Clinical Management of Disease States
  TAGS:
  Bronchopulmonary Fistula
4. Special Considerations in Thoracic Anesthesia
  Isolated Lung Ventilation

#### O. Cardiac Anesthesia

3. Clinical Management of Disease States
  TAGS:
  Pericardial Effusion/Tamponade
4. Special Considerations in Cardiac Anesthesia
  TAGS:
  Unintended Intraoperative Awareness

#### P. Vascular Anesthesia

4. Special Considerations in Vascular Anesthesia
  TAGS:
  Aortic Clamping

#### Q. Regional Anesthesia

6. Truncal Regional Anesthesia
  TAGS:
  Abdominal Wall Blocks: TAP, Ilioinguinal, Iliohypogastric, Rectus Sheath
  Posterior Truncal Blocks: Quadratus Lumborum, Erector Spinae, Retrolaminar
  Truncal Block Anatomy and Sonoanatomy

### VI. SPECIAL PROBLEMS OR ISSUES IN ANESTHESIOLOGY

#### B. Organ Donors

1. Pathophysiology
2. Clinical Management
  TAGS:
  Criteria for Brain Death
  Donation After Cardiac Death

#### C. Radiologic Procedures

3. Anesthesia in Locations Outside the Operating Room
  TAGS:
  Airway Management
  Monitoring Requirements

#### E. Ethics, Practice Management and Medicolegal Issues

2. Ethics
  TAGS:
  Do Not Resuscitate Orders and Advance Directives
  End-of-Life Issues (Withholding/Withdrawal)
  Jehovah’s Witness Patient Care

### IX. CRITICAL CARE MEDICINE

#### A. Basic Pathophysiology

2. Cardiovascular
  TAGS:
  Cardiac Contusion
  Pulseless Electrical Activity
  Shock States
  Trauma
3. Pulmonary
  TAGS:
  Bronchopleural Fistula
  Chest Trauma
  Pleural Effusion
  Tracheal Disruption
5. Hematologic/Oncologic
  TAGS:
  Carboxyhemoglobin
  Coagulopathies
  Disseminated Intravascular Coagulation
  Fibrinolysis
  Methemoglobin
11. Acid-base and Electrolyte Abnormalities
  TAGS:
  Acid-base Abnormalities
  Calcium
  Chloride
  Electrolyte Abnormalities
  Magnesium
  Metabolic
  Mixed
  Phosphorus
  Potassium
  Respiratory
  Sodium

#### B. Critical Illness Diagnosis and Management

1. Central Nervous System
  TAGS:
  Head Injury, Closed or Open
  Hemorrhagic (Subarachnoid, Subdural, Epidural Hematoma)
  Intracranial Pressure Measurement
  Intracranial Pressure-Controlling Medications
  Jugular Venous Saturation
  Spinal Cord Injury
  Steroids
  Stroke
  Subarachnoid, Subdural, Epidural Hematoma
2. Cardiovascular
  TAGS:
  Cardiac Contusion
  Cardiac Ultrasound (Transthoracic Echocardiograms, Transesophageal Echocardiograms)
  Other Bedside Ultrasound
  Pericardial Effusion
  Pericardiocentesis
  Tamponade
3. Pulmonary
  TAGS:
  Chest Trauma
  Pleural Drainage and Evacuation
  Pleural Effusion
  TRALI
  Ultrasound
4. Renal
  TAGS:
  Continuous Renal Replacement Therapies Including Ultrafiltration
5. Hematologic/Oncologic
  TAGS:
  Acquired
  Anemia
  Anticoagulants
  Antiplatelet Agents
  Carboxyhemoglobin
  Coagulation Studies
  Coagulopathies
  Disseminated Intravascular Coagulation
  Fibrinolysis
  Methemoglobin
  Reversal Agents
  Transfusion and Factor Replacement
  Vitamin K Dependent
7. Endocrine
  TAGS:
  Cerebral Salt Wasting
  Cushing Syndrome
  Diabetes Insipidus Including Central, Nephrogenic
  SIADH
  Vasopressins/DDAVP
8. Gastrointestinal
  TAGS:
  Abdominal Compartment Syndrome
  Blood Product Selection and Administration

#### C. Specialized Areas

2. Burns
  TAGS:
  Airway Management
  Antimicrobials
  Complications
  Electrical Burns
  Fluids and Resuscitation
  Inhalation Injury
  Management
  Other Therapies (Hyperbaric, Pharmacologic, Surgical)
3. Disaster Management
  TAGS:
  Biologic, Chemical, and Nuclear Exposures
  Epidemic
4. Drowning, Fatal, Near drowning
  TAGS:
  Fresh Water
  Salt Water
8. Life Support and Resuscitation
  TAGS:
  Other
13. Procedures
  TAGS:
  Chest Tubes
  Intraosseous

### XI. PEDIATRIC ANESTHESIOLOGY

#### B. Organ-Based Basic and Clinical Sciences

3. Central and Peripheral Nervous Systems
  TAGS:
  Intracranial Pressure and Blood Flow

#### C. Clinical Subspecialties

5. Ophthalmology
  TAGS:
  Trauma
7. Trauma and Burns
  TAGS:
  Anesthetic and Pain Management of the Burn Patient
  Burns
  Dressing Changes
  Fluid Resuscitation and Calculating Burn Surface Area
  Hypothermia and Submersion Injury
  Incidence, Patterns, Implications of Abuse
  Inhalation Injuries/Airway Management
  Management of the Polytrauma Victim
  Trauma
  Types, Mechanisms, Locations and Implications of Injuries

#### D. Clinical Science of Anesthesia

2. General Considerations of the Perioperative Period
  TAGS:
  Transfusion Therapy and Blood Conservation Techniques
