# Supplementary material for: Training Anesthesiology Residents to Care for the Traumatically Injured in the United States
Source: Anesth Analg. 2023 Apr 14;136(5):861–76. doi: 10.1213/ANE.0000000000006417 (PMC10079293; doi:10.1213/ANE.0000000000006417)
Supplement: Supplementary file 1 [file ane-136-861-s001.docx]

Supplemental Table 1. Representative Content Deemed Trauma Related from the Initial Certification in Anesthesiology Content Outline Which is Not Found in Section II.D.9 Trauma Anesthesia

| Heading | General Theme | Specific Examples |
| --- | --- | --- |
| Anatomy | - Topographical Anatomy as landmarks | - Cricothyroid Membrane, Jugular Veins, etc. |
|  | - Radiological and Ultrasound Anatomy | - Chest, Brain, Spine, Neck, etc. |
| Physics, Monitoring, Anesthesia Delivery Devices | - Physics | - Carbon Monoxide, etc. |
|  | - Instrumentation | - Fluid Warmers, Echocardiography, Point-of-Care Ultrasound, etc. |
|  |  | - Intracranial Pressure, Coagulation, etc. |
|  | - Monitoring | - Effects on CNS, etc. |
| Pharmacology | - Anesthetics-Gases and Vapors |  |
|  | - Oral Anticoagulants and Anti-platelets | - Mechanism of Action, Effects on monitoring, Implications for Regional Anesthesia, etc. |
| Preoperative Patient Evaluation | - Preparation for Anesthesia | - Full Stomach Status, Implications for Airway Management, etc. |
| General Anesthesia | - Airway Management | - Identification of Difficult Airway, Techniques for Managing Airway, Surgical Airway, Endobronchial Intubation, etc. |
| Common Complications |  | - Hypothermia |
| Central & Peripheral Nervous System | - Physiology, Anatomy | - Cerebral Perfusion Pressure, Cerebral Blood Flow Autoregulation, Subdural and Subarachnoid Spaces, etc. |
|  | - Clinical Science | - Glasgow Coma Scale, Spinal Shock, Fluid Management, etc. |
| Respiratory System | - Ventilation, Diffusion, Blood Gas | - Hypoxic Pulmonary Vasoconstriction, Apneic Oxygenation, Blood Gas Interpretation, etc. |
|  | - Clinical Science | - Chest, Bronchial, and Upper Airway Trauma, etc. |
| Cardiovascular System | - Physiology | - Venous Return, Blood Pressure Monitoring, Microcirculation, etc. |
|  | - Pathology | - Tamponade, PE, Shock States, etc. |
| Hematologic System | - Transfusions | - Indications, Blood Components, Reactions, Complications, MTP, etc. |
|  | - Disorders | - Coagulopathy, Fibrinolysis, etc. |
| Regional Anesthesia |  | - Relevant Anatomy, Management, etc. |
| Critical Care | - Shock | - Etiology, Sepsis, Multiple Organ Dysfunction, etc. |
|  |  | - Drowning |
| Special Problems | - Organ Donation | - Management, Brain Death Criteria, etc. |
